# A Rare Case of Autoimmune Pancreatitis in a 9-Year-Old Male Patient: A Comprehensive Diagnosis and Successful Treatment

**DOI:** 10.1155/crgm/5564385

**Published:** 2024-12-05

**Authors:** Hadi Farhat, Christie Dib, Yehya Tlaiss, Ayman Tabcheh, Pierre Hani

**Affiliations:** ^1^Department of General Surgery, Faculty of Medicine and Medical Sciences, University of Balamand, Dekwaneh, Beirut, Lebanon; ^2^Department of Gastroenterology, Faculty of Medicine and Medical Sciences, University of Balamand, Dekwaneh, Beirut, Lebanon

**Keywords:** autoimmune, corticosteroid therapy, pancreatitis, pediatric patient

## Abstract

Autoimmune pancreatitis (AIP) is a rare and complex condition that can be difficult to identify due to its resemblance to malignancies. This case report presents a unique instance of AIP in a 9-year-old male patient who presented with painless jaundice and elevated liver function test results. His symptoms were persistent even after previous common bile duct stent placement, requiring additional investigation. The possibility of AIP was raised by further serological tests and imaging examinations. The diagnosis was then confirmed by multiple characteristic findings revealed through history, imaging, clinical examination, histology, and lab results. Treatment was initiated with corticosteroids, which resulted in a complete resolution of symptoms and remarkable recovery. This case emphasizes the significance of including AIP in the differential diagnosis of pancreatic disorders, even in pediatric patients.

## 1. Introduction

Autoimmune pancreatitis (AIP) is a rare chronic fibroinflammatory disease of the pancreas [1]. The International Consensus Diagnostic Criteria 2010 (ICDC) defined AIP as a distinct subtype of pancreatitis characterized by obstructive jaundice with or without pancreatic masses, lymphoplasmacytic infiltrate and fibrosis, and a significant response to steroids. AIP can be further divided into two categories: Type 1 and Type 2 [2].

Type 1 AIP, previously called lymphoplasmacytic sclerosing pancreatitis (LPSP), is a local pancreatic manifestation of an IgG4-related systemic disease [2, 3]. It is mainly observed in Asian populations and tends to affect male patients over 50 years of age [3]. The clinical presentation mainly consists of patients with obstructive jaundice and chronic, mild, or recurrent abdominal pain. Imaging, using contrast-enhanced computed tomography (CT), often reveals diffuse swelling of the pancreas (sausage-shaped pancreas) with segmental enlargements [4, 5].

Type 2 AIP, also known as idiopathic duct-centric pancreatitis (IDCP), is a more recent classification of AIP that tends to affect young Caucasians, including children [2]. Patients clinically present with acute pancreatitis, jaundice, abdominal pain, or features suggestive of inflammatory bowel disease, most commonly ulcerative colitis [2, 5, 6].

The international standards for diagnosing AIP include the Mayo Clinic's HISORt criteria, which are based on histology, imaging, serum IgG4 levels, additional organ involvement, and response to treatment with steroids, as well as the ICDC [7]. Regardless, reaching a definitive diagnosis of AIP, especially Type 2 AIP, remains a challenge since the condition frequently manifests as a pancreatic mass mimicking pancreatic cancer [7].

In this context, we report a unique case of a 9-year-old male patient who first presented with painless jaundice and elevated liver function tests. Despite the previous implantation of a common bile duct stent, these symptoms persisted, prompting a thorough medical assessment. Informed consent from the patient and his parents was obtained prior to writing this case.

## 2. Case Report

A 9-year-old male patient sought medical attention due to painless jaundice and elevated liver function test results. These symptoms recurred despite a previous common bile duct stent placement. An initial CT scan revealed diffuse involvement resulting in a characteristic “sausage-shaped” pancreas. The initial lab results indicated elevated levels of both direct and indirect bilirubin, increased gamma-glutamyl transferase, elevated alkaline phosphatase, heightened aspartate aminotransferase, and alanine aminotransferase, along with mild anemia. Lipase and C-reactive protein levels were within the normal range (Table 1).

Our differential diagnosis included biliary colic, pediatric cholecystitis, pediatric gastroenteritis, acute pancreatitis, and peptic ulcer disease. However, upon reviewing the patient's history, there were no reports of postprandial abdominal pain or waxing and waning discomfort. Moreover, during the physical examination, right upper quadrant pain was absent, and the Murphy sign was negative. An abdominal ultrasound was conducted, revealing no evidence of gallstones or inflammation of the gallbladder, effectively ruling out biliary colic and acute cholecystitis. Additionally, there were no symptoms of diarrhea, nausea, or vomiting noted, and the patient's vital signs remained stable without any preceding fever, diminishing the likelihood of gastroenteritis.

Furthermore, the patient had no history of NSAID use or alcohol abuse, making peptic ulcer disease unlikely. Subsequently, acute pancreatitis emerged as a primary consideration, prompting a detailed exploration of various etiologies of pancreatitis in pediatric patients.

Since there were no observed gallstones on abdominal ultrasound, biliary pancreatitis was conclusively ruled out. Furthermore, the absence of a history of alcohol abuse or medication/drug use known to trigger pancreatitis aided in eliminating alcoholic and drug-induced pancreatitis as potential causes. Notably, there were no indications of congenital pancreatic abnormalities, including pancreatic divisum, or any history of cystic fibrosis. A thorough clinical examination allowed us to dismiss viral etiologies like mumps, and the patient's vaccination records were up to date. There was also no history of abdominal trauma.

**Figure 1:**
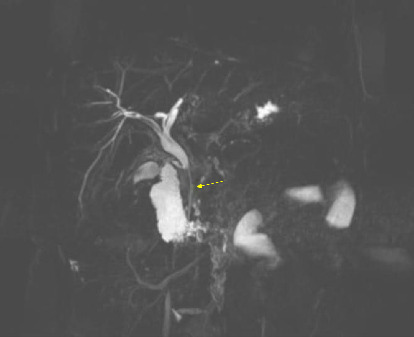
MRCP showing a stent in the common bile duct (yellow arrow) with a long segment of narrowing in the head of the pancreas without dilatation of the pancreatic duct.

**Figure 2:**
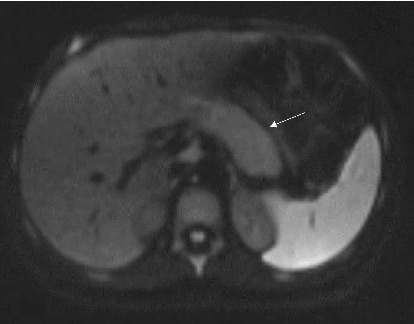
Diffusion showing mild edema of the pancreas with a featureless appearance (white arrow).

**Figure 3:**
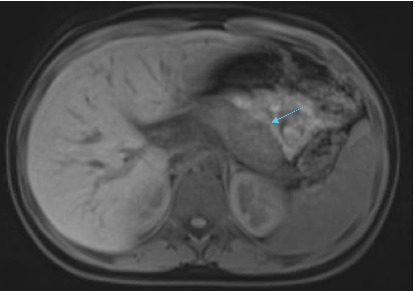
T1 with fat saturation showing the absence of the normal high signal intensity of the pancreas (blue arrow).

On April 16, 2022, a magnetic resonance cholangiopancreatography (MRCP) without contrast was performed, confirming the presence of a biliary stent, but it also revealed narrowing at the level of the pancreatic head and again confirmed the characteristic “sausage-shaped” pancreas. Furthermore, there was a diffuse loss of normal signal intensity in the pancreas, with mild enlargement, yet no evidence of a mass. The narrowing in the distal common bile duct extended approximately 3 cm along the intrapancreatic portion (Figures 1, 2, 3).

Our differential diagnosis for common bile duct stricture included primary sclerosing cholangitis, cholangiocarcinoma, IgG-4-related sclerosing cholangitis, AIP, and benign biliary stricture. The MRCP results helped us rule out primary sclerosing cholangitis. Benign biliary stricture is unlikely due to a lack of history of trauma, previous surgery, or ischemia. Labs were subsequently ordered to rule out the other etiologies.

Autoimmune serology and tumor marker tests were conducted, revealing normal values for alpha1-antitrypsin, immunoglobulin (IgG)-4, antinuclear antibodies, antimitochondrial antibodies, endoplasmic reticulum antibodies, antinuclear cytoplasmic antibodies, antismooth muscle antibodies, alpha-fetoprotein, CA 19-9, and CEA (Table 2). The normal IgG-4 value ruled out IgG-4-related sclerosing cholangitis and Type 1 AIP, and the normal levels of CA19-9 and CEA made cholangiocarcinoma unlikely. This puts Type 2 AIP high on the differential. Since autoimmune serology did not provide a conclusive diagnosis, an endoscopic ultrasound (EUS) was scheduled for April 20, 2022.

**Figure 4:**
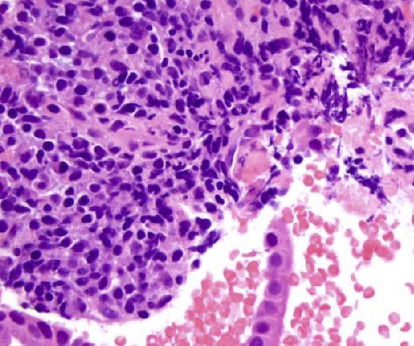
Histology result of pancreatic biopsy after endoscopic ultrasound.

During the EUS, a comprehensive examination of the pancreas revealed a granular, heterogeneous, hypoechoic appearance with the presence of hyperechoic septa. No discernible tumors were observed. Additionally, the pancreas displayed edema, assuming a distinctive “sausage” shape, a characteristic associated with AIP. The common bile duct exhibited a thickened, hyperechogenic wall, measuring 6 mm in thickness, and contained a plastic prosthetic stent. Fine-needle aspiration with multiple biopsy specimens of the pancreas was taken to rule out underlying malignancy, especially in the setting of common bile duct stenosis, and to check for AIP which was high in the differential diagnosis. The biopsy findings effectively excluded malignancy, revealing a fibroinflammatory process predominantly affecting pancreatic ducts and acinar atrophy of the pancreatic head which is consistent with active pancreatitis (Figure 4). A confirmatory element in our diagnosis of AIP was evident through the presence of neutrophilic infiltration in both medium-sized and small ducts.

A trial of corticosteroids, specifically prednisone at a 20 mg dosage, was initiated and yielded a remarkable resolution of the patient's symptoms. This treatment regimen was continued for a duration of 6 weeks, during which no signs of symptom recurrence were observed which confirmed the diagnosis of Type 2 AIP. The weight and height of the patient were 28 kg and 133 cm at the time of initiation of the treatment. This dosing was adopted based on the international consensus for the treatment of AIP which states that the initial dose of prednisone should be 0.6–1.0 kg mg/kg/day and that 20 mg/day is generally necessary to induce remission [8]. The patient was followed up after 2 and 6 weeks, revealing resolution of his jaundice and an improved clinical presentation. Follow-up laboratory tests showed normal lipase levels and liver function tests.

**Figure 5:**
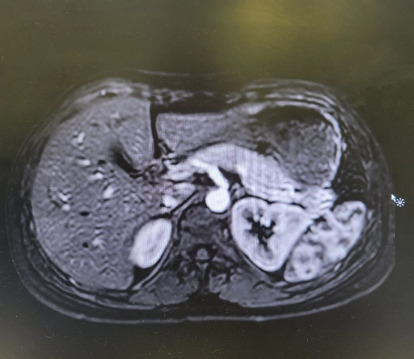
MRI showing pancreas after full recovery.

On June 28, 2022, a follow-up MRCP was conducted, revealing the complete resolution of the prior common bile duct narrowing near the pancreatic head. The pancreatic duct now appeared clearly delineated without any signs of constriction. Additionally, the mild pancreatic enlargement indicative of AIP that was previously observed had fully resolved (Figure 5). Subsequently, on July 11, 2022, the common bile duct stent was safely removed, and the patient was discharged after making a full recovery.

## 3. Discussion

The term “AIP” was first introduced by Yoshida et al. in 1995 to describe an autoimmune disease responsive to steroid treatment [9]. As mentioned earlier, AIP can be categorized into two subtypes: Type 1 AIP (LPSP) and Type 2 AIP (IDCP) [2, 4]. In Japan, the prevalence of AIP is quite rare, with only 0.82 cases per 100,000 individuals. Type 1 AIP is the most common worldwide, while Type 2 AIP is more prevalent in Europe and North America [7].

A global study involving 1064 patients found that the average age at diagnosis was 61.4 years for Type 1 AIP and 39.9 years for Type 2 AIP, with a significantly higher proportion of male patients in Type 1 (77% vs. 55%) [10]. However, our case involves a 9-year-old pediatric patient, which is highly unusual. Estimating the true incidence of pancreatitis in the pediatric population is challenging, especially since most of the literature consists of case reports or small case series. AIP in children is often underdiagnosed and necessitates a high level of clinical suspicion.

Diagnosing AIP has long posed a challenge, as evidenced by numerous published reports. A notable case study by Ravanbakhsh et al. in 2023 sheds light on this complexity [11]. They detailed the case of a 10-year-old female with symptoms mirroring those of our patient. Their differential diagnosis, including pancreatic malignancy and biliary stricture, paralleled our own clinical considerations. Employing labs and imaging techniques such as MRCP and ultrasound guided the diagnosis. Ultimately, histological confirmation was pivotal, leading to the initiation of corticosteroid therapy and successful treatment.

The approach taken by other authors, such as Hasosah et al. and Ramos et al., parallels the methodology employed in our case [12, 13]. Hasosah et al. documented a case involving a 10-year-old female, while Ramos et al. focused on a 16-year-old female, both diagnosed with AIP. These studies employed comparable laboratory investigations and imaging modalities, emphasizing the consistency in diagnostic strategies across cases. Furthermore, corticosteroid therapy emerged as the cornerstone treatment in these instances. Evidently, reaching a conclusive diagnosis posed a significant challenge in each scenario.

The Japan Pancreas Society has established diagnostic criteria for distinguishing AIP from other forms of pancreatitis and pancreatic cancer [14]. These criteria are based on a comprehensive evaluation of imaging, laboratory tests, and histopathological findings. Various imaging modalities can be utilized, including CT scans, ultrasound, EUS, and endoscopic retrograde cholangiopancreatography (ERCP).

An abdominal CT scan of AIP patients typically reveals the distinctive “sausage shape” pattern along with signs of atrophy or inflammation. Additional features observed include diffusely decreased enhancement of the pancreas, a capsule-like rim, and bile duct enhancement. Ultrasound examinations can unveil stenosis in both intrahepatic and extrahepatic ducts, while ERCP may show narrowing and irregularity of the pancreatic duct. EUS, on the other hand, often demonstrates hypoechoic pancreatic enlargement, bile duct thickening, and peripancreatic hypoechoic margins [15]. In our case, we employed a combination of imaging techniques, specifically a CT scan, MRCP, and EUS with biopsy, all of which consistently revealed findings consistent with those documented in the existing literature.

The most prominent laboratory abnormality in the majority of cases is hyperbilirubinemia, notably elevated direct bilirubin levels [16], a condition that our patient also presented. Hypergammaglobulinemia, in combination with elevated IgG-4 levels, is a characteristic feature in 68% of patients [16]. Elevated IgG-4 levels play a crucial role in differentiating AIP from pancreatic cancer, with a remarkable 95% sensitivity and 97% specificity [17]. In the case of Type 1 AIP, positive IgG-4 levels (> 150 mg/dL) are typically indicative of multi-organ involvement, although instances of IgG-4-negative Type 1 AIP can also be found. In contrast, Type 2 AIP patients typically exhibit low IgG-4 levels (< 128 mg/dL), suggesting a localized pancreatic disease [15]. Our patient exhibited negative IgG-4 levels making our diagnosis even more challenging.

The clinical presentation of AIP exhibits substantial variation within the existing literature. In our specific case, the patient experienced recurrent episodes of pancreatitis and sought medical attention with painless jaundice accompanied by elevated liver function tests. While abdominal pain attacks have been documented in numerous cases [18], it is noteworthy that over half of the patients manifest symptoms of obstructive jaundice resulting from bile duct stenosis [18], mirroring the presentation in our case.

Type 1 and Type 2 AIP exhibit distinct histological characteristics. In Type 1 AIP, histological examination typically reveals a periductal lymphoplasmacytic infiltrate with obliterative phlebitis and storiform fibrosis, alongside a notable presence of IgG4-positive cells (usually exceeding 10 cells per high-power field [hpf]) [15]. The lymphoplasmacytic infiltrate is primarily composed of CD8-and CD4-positive lymphocytes. On the other hand, Type 2 AIP presents with ductal granulocyte epithelial lesions when examined histologically [15]. Our biopsy showed fibroinflammatory process predominantly affecting pancreatic ducts and the presence of neutrophilic infiltration in both medium-sized and small ducts. This is in accordance to the literature.

Our diagnosis was further confirmed by the patient's significant and positive response to corticosteroid treatment, which is consistent with the diagnostic criteria for AIP outlined in both the Mayo Clinic guidelines [19] and the guidelines provided by the Korean Society of Pancreatobiliary Diseases [20]. Notably, AIP patients typically exhibit rapid improvement within 2 weeks of initiating corticosteroid therapy, a response that aids in distinguishing AIP from other potential pancreatic causes [18]. The standard starting dose is 1 mg/kg/day, gradually tapering by 2.5–5 mg per week. In some cases, a long-term maintenance dose of 5–10 mg/day may be necessary [18].

## 4. Conclusion

In summary, this case report illustrates a unique case of AIP in a 9-year-old patient, highlighting the challenges involved in diagnosing this condition and the efficacy of corticosteroid treatment. It is imperative to stress that, despite its rarity, AIP should always be taken into account in the differential diagnosis, particularly when assessing a pediatric patient presenting with painless jaundice. Furthermore, the quick remission that was observed within just 6 weeks emphasizes how crucial it is to identify pediatric cases early on and take prompt action. Sharing such cases advances medical knowledge by directing future therapeutic plans and ensuring better results for patients with this form of disease.

## Figures and Tables

**Table 1:** Patient lab tests on initial presentation.

Test	Result	Reference range
Amylase	40 U/L	26–115
Lipase	28 U/L	22–51
Bilirubin, direct	3.8 mg/dL	0.01–0.12
Indirect bilirubin	4.09 mg/dL	0.1–0.7
Alkaline phosphatase	762 U/L	134–315
Gamma glutamyl transferase	29 U/L	5.0–12
Aspartate aminotransferase	40 U/L	21–39
Alanine aminotransferase	46 U/L	12.0–27
C-reactive protein	4 mg/L	0–10
Calcium	10.3 mg/dL	8.8–10.8
Creatinine	0.54 mg/dL	0.28–0.56
Ferritin	56 ng/mL	10.29–55.84
Iron	78 μg/dL	17–129
Protein	66 g/L	66–81
Albumin	39 g/L	38–48
Globulin	27 g/L	23–30
Ratio (albumin/globulin)	1.44	1.5–1.7
Hemoglobin	10.2 g/dL	12–15
Hematocrit	30.7%	35–49
Mean corpuscular volume	75 fL	6–11

**Table 2:** Autoimmune and tumor marker serology that was ordered for the patient.

Test	Result	Reference range
Alpha1-antitrypsin	187.69 mg/dL	90–200
IgG-4	0.435 g/L	8–10 years: 0.019–0.932
Alpha-fetoprotein	1.4 ng/mL	0.8–7.3
CA 19-9	25 U/mL	Negative < 34
CEA	< 0.5 ng/mL	Negative < 2.5
Antinuclear antibodies	Negative < 1/100	Negative < 1/100
Antimitochondrial antibodies	Negative < 1/100	Negative < 1/100
Endoplasmic reticulum antibodies	Negative < 1/100	Negative < 1/100
Perinuclear antineutrophil cytoplasmic antibodies	Negative < 1/100	Negative < 1/100
Cytoplasmic antineutrophil cytoplasmic antibodies	Negative < 1/100	Negative < 1/100
Antismooth muscle antibodies	Negative < 1/100	Negative < 1/100

## Data Availability

The underlying data supporting the results of our study are available upon request. Please contact corresponding author Yehya Tlaiss at yehyatlaiss@hotmail.com for access. We aim to ensure the transparency and reproducibility of our research findings, and we are committed to providing access to the data underlying our results to facilitate further exploration and validation by the scientific community.
